# A Statewide Recommendation for Opt-Out Syphilis Screening in Nonpregnant Adults: Rationale and Evidence-to-Decision Framework

**DOI:** 10.1016/j.focus.2026.100509

**Published:** 2026-05-05

**Authors:** Elizabeth H. Lees, Elizabeth M. Dufort, Nicholas B. Lehnertz, Khalid Bo-Subait, Cindy K. Lind Livingston, Marisela D. Babcock, Alice Lehman, M. Hassan Murad, Ruth Lynfield

**Affiliations:** 1 Mayo Clinic Division of Public Health, Infectious Disease, and Occupational Medicine, Rochester, Minnesota; 2 Minnesota Department of Health, Saint Paul, Minnesota; 3 Division of Adult and Pediatric Infectious Diseases, University of Minnesota, Minneapolis, Minnesota

**Keywords:** Syphilis, screening methods, opt-out, infection prevention, public health practice

## Abstract

**Introduction:**

Syphilis rates have increased substantially across the U.S. over the past decade. In response, the Minnesota Department of Health reviewed and updated its screening recommendations.

**Methods:**

A scoping review was performed to identify published manuscripts and guidelines on syphilis screening. The Minnesota Department of Health reviewed national and local syphilis data (2016–2024), published evidence, and screening approaches from other jurisdictions. The final recommendation was based on decisional factors derived from the GRADE evidence-to-decision framework and an adaptation of evidence-to-decision that is specific to screening. Multiple factors were considered, including disease burden, treatment availability, screening accessibility, implementation feasibility, and access to health care.

**Results:**

Evidence shows that early detection and treatment of syphilis are highly effective. Opt-out screening models achieve greater uptake and case detection than risk-based screening approaches. On the basis of the findings, the Minnesota Department of Health endorsed a new universal, opt-out syphilis screening recommendation for all nonpregnant individuals aged 18–49 years.

**Conclusions:**

Universal opt-out screening for syphilis has the potential to reduce the syphilis disease burden in Minnesota. Other jurisdictions may wish to adopt similar policies and may do so by modeling this approach.

## Highlights

• Syphilis rates have increased in the U.S over the past decade.
• Most screening guidelines for syphilis in the U.S are risk based.
• Opt-out screening has been implemented for other sexually transmitted infections.
• Individual states may wish to implement their own syphilis screening guidelines.
• Opt-out screening guidelines are one consideration to increase syphilis detection.

## INTRODUCTION

Syphilis cases in the U.S. have risen drastically over the last decade; from 2015 to 2024, the U.S. saw a greater than 150% increase from 74,215 cases to 186,301.^1^ Current recommendations endorsed by the U.S. Preventive Services Task Force (USPSTF) in 2022 recommend syphilis screening for asymptomatic, nonpregnant adolescents and adults who are at increased risk for syphilis infection and for all pregnant women.^2^ Similarly, the Centers for Disease Control and Prevention (CDC) recommend syphilis screening for those at increased risk for syphilis owing to risk factors, including history of incarceration or transactional sex work, race/ethnicity, age, and gender identity; those who are men who have sex with men (MSM); persons living with HIV; gender-diverse individuals based on sexual exposures; and geography.^3^ The CDC defines geographic risk as counties with rates of primary and secondary syphilis of 4.6 per 100,000 people or greater among females aged 15–44 years and recommends that syphilis screening be offered to sexually active people aged 15–44 years in these counties.^4^ Although provisional data from 2024 show a modest decline in the rates of overall syphilis, congenital syphilis cases have continued to rise, with nearly 4,000 U.S. cases reported in 2024.^1^ Although the USPSTF and CDC recommend screening once early in pregnancy and additionally on the basis of risk factors, the American College of Obstetricians and Gynecologists updated their screening recommendations in 2024 for all pregnant people to be screened for syphilis at the first prenatal visit, followed by universal rescreening during the third trimester and at birth.^5^, ^6^, ^7^, ^8^

The Minnesota Department of Health (MDH) has historically aligned with national sexually transmitted infection screening guidelines from the CDC. However, from 2014 to 2023, there was a 166% increase in overall syphilis cases in Minnesota, and congenital syphilis cases were at their highest number in over 40 years.^9^ Owing to the continued rise in syphilis and congenital syphilis cases, MDH expanded on national guidelines to issue new statewide guidance on syphilis screening. First, in January 2024, MDH recommended syphilis screening 3 times during each pregnancy (at the first prenatal encounter, during the third trimester, and at delivery).^10^ Then, in September 2024, MDH recommended universal syphilis screening for asymptomatic nonpregnant adults aged 18–49 years, using an opt-out approach, at least once as part of routine health care.^11^ Traditional risk-based, opt-in screening can be limited by incomplete history taking, patient failure to disclose, and provider discomfort; thus, the new population-wide, opt-out recommendation is intended to address these barriers.^12^

A number of other public health entities have responded to the syphilis epidemic through more comprehensive syphilis screening recommendations for nonpregnant people. Washington state was early to adopt universal screening for sexually active individuals in January 2021 in response to the increasing syphilis cases among heterosexual individuals and newborns in the state, especially those experiencing homelessness.^13^ New Mexico responded to its statewide syphilis epidemic in 2022 by implementing universal testing for all individuals aged 18–50 years.^14^ By 2024, primary and secondary syphilis rates had dropped by 31%, and congenital syphilis rates had decreased by 19.6% in the state.^14^ In 2024, California began recommending screening for all sexually active persons aged 15–44 years at least once in their lifetime and annually thereafter.^15^ It is notable that Minnesota is home to 11 federally recognized American Indian tribes, which make up 2%–3% of the statewide population.^16^ In July 2023, the Indian Health Services issued a letter to tribal leaders recommending annual syphilis testing for persons aged 13–64 years, expanded further in February 2024 to recommend annual syphilis testing for persons aged ≥13 years.^17^ In considering updated syphilis screening in Minnesota, MDH evaluated these other jurisdictional approaches to develop its own state-level guidance.

## METHODOLOGY

In expanding upon the current national guidelines, the team at MDH began with a scoping review of the Medline/PubMed database as well as an internet search for published reviews and guidelines on the relevant topics. The team did not conduct a formal systematic review because of the wide availability of existing publications.

The team at MDH considered decisional factors that can be mapped to 2 existing frameworks for decision making in the context of screening: the GRADE (Grades of Recommendations, Assessment, Development, and Evaluation) EtD (Evidence-to-Decision) framework presented by Alonso-Coello et al. and a more screening-specific framework presented by Murad et al., which emphasizes importance, natural history, difference in management, available treatment, difference in outcomes, accuracy, cost, acceptability, and implementation.^18^^,^^19^ The MDH decision-making process is described step by step to offer a model for other public health entities and communities pursuing statewide screening recommendations. This study was conducted ethically and exempted from IRB approval given its use of deidentified, publicly available data.

### Natural History and Importance

Once considered nearly eliminated in the U.S., syphilis has experienced a significant resurgence, with national rates in 2023 reaching the highest level recorded since 1950.^20^ This trend is reflected in Minnesota, where the increase in cases has affected males and females across multiple age groups, including newborns with congenital syphilis. Left untreated in adults, syphilis can cause complications ranging from cardiac disease to neurologic deficits, blindness, deafness, and psychosis.^21^

**Figure 1:**
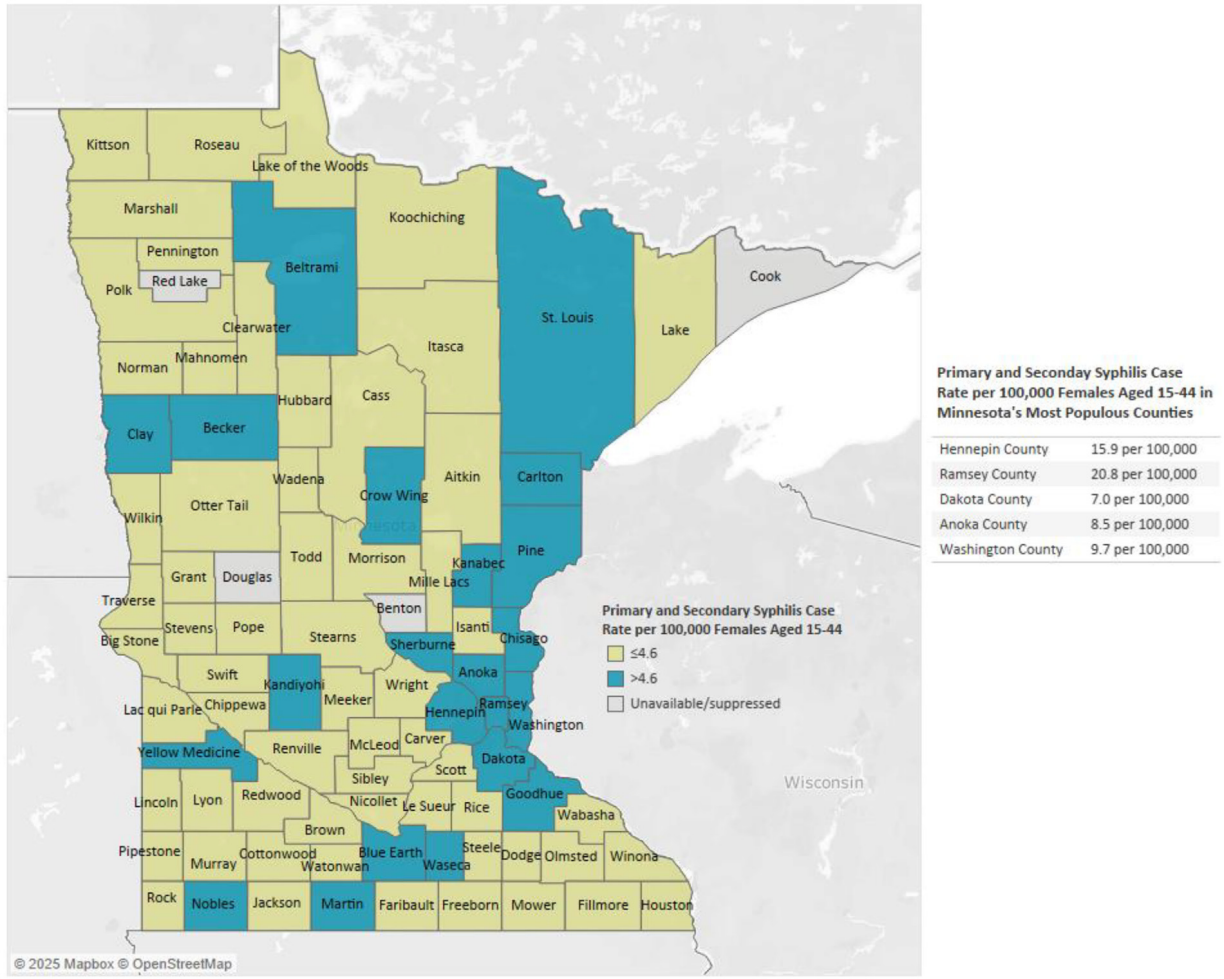
County-level map of primary and secondary syphilis rates among females aged 15–44 years per 100,000 people, Minnesota 2023. *Note*: The map highlights Minnesota counties in 2023 where screening is recommended by geography on the basis of CDC guidelines. This threshold to screen all sexually active males and females is determined by syphilis rates >4.6 per 100,000 people among females aged 15–44 years. The table displays the rates in the 5 most populous Minnesota counties. CDC, Centers for Disease Control and Prevention.

One of the core objectives listed by the HHS *Healthy People 2030* initiative is to decrease primary and secondary syphilis in females aged 15–44 years to fewer than 4.6 cases per 100,000.^22^ Currently, 46% of counties in the U.S. fail to meet this metric and are considered high-risk geographic counties.^23^ In these high-risk counties, the CDC has recommended screening all sexually active people aged 15–44 years for syphilis.^23^ There are 22 geographically dispersed Minnesotan counties, including the top 5 most populous counties in the state, that do not meet this *Healthy People 2030* syphilis objective (Figure 1).^9^ Hence, per the CDC’s geographic risk-based screening, a majority of the population in Minnesota should be screened for syphilis on the basis of geography of residence alone. However, it can be challenging to implement geographic risk-based recommendations given the complexity and the fact that areas where individuals choose to live, work, move, and have sex often cross county lines.

**Figure 2:**
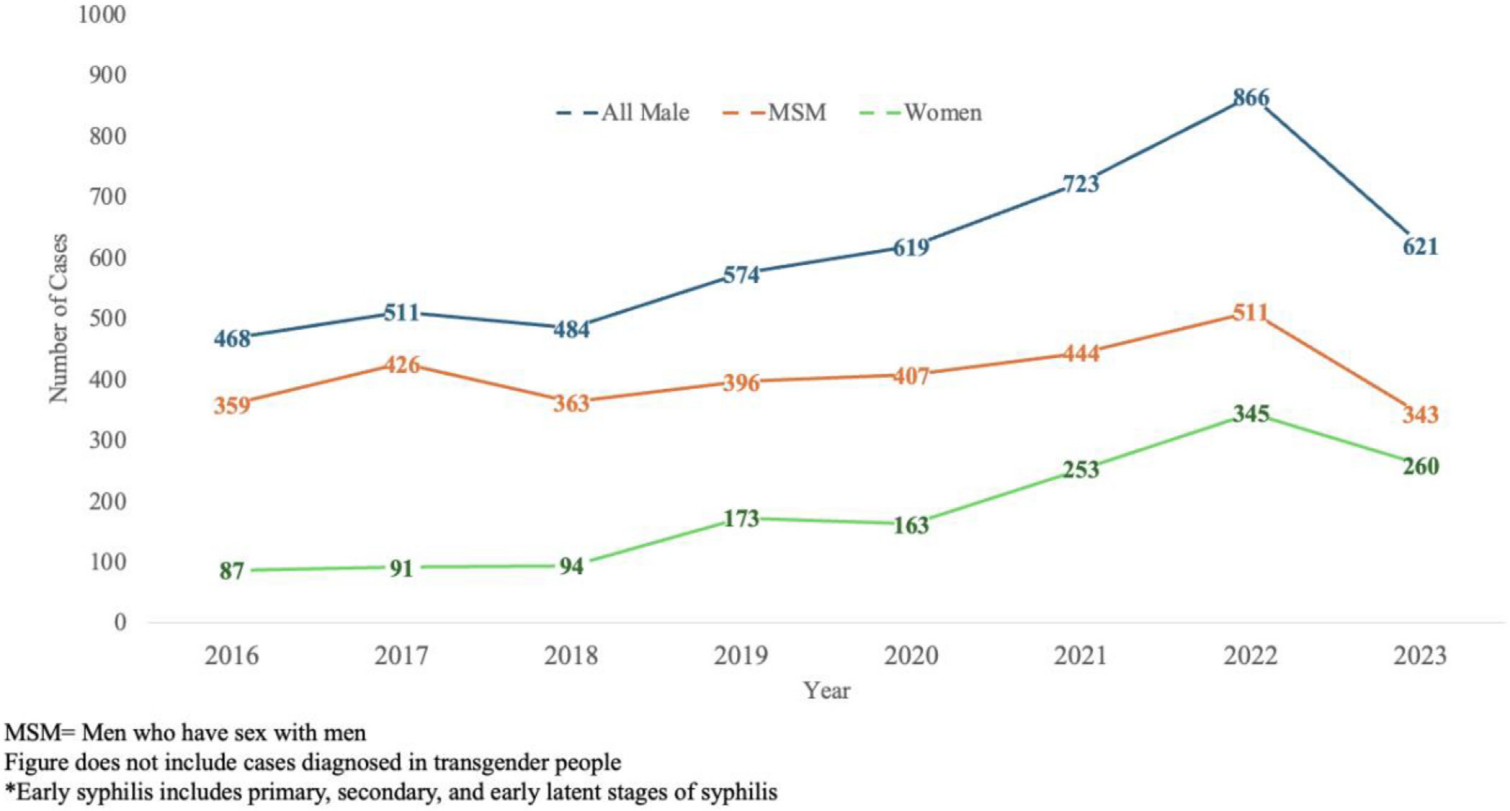
Number of early syphilis^a^ cases by gender and MSM, Minnesota, 2016–2023. *Note*: Figure does not include cases diagnosed in transgender people. ^a^Early syphilis includes primary, secondary, and early latent stages of syphilis. MSM, men who have sex with men.

In 2023, the overall rate of syphilis infections among Minnesota residents of all ages was 28.4 per 100,000. The rate of primary and secondary syphilis in Minnesota was 8.8 cases per 100,000 persons, with a rising trend among females.^9^ The rise of syphilis in females parallels an increase among men who have sex with women, suggesting a growing heterosexual syphilis epidemic in Minnesota (Figure 2).^9^ Early syphilis (the combined number of primary, secondary, and early latent) cases in females living in Minnesota increased by 534% in the past decade, which correlates with the increased number of congenital syphilis cases in Minnesota over this timeframe. There were 26 congenital cases, including 3 stillbirths, in Minnesota in 2023.^9^

### Available Treatment

Treatment for syphilis consists of benzathine penicillin G as a first-line agent or doxycycline as an alternative for nonpregnant patients. Early syphilis has a treatment success rate of 90%–100% with a single intramuscular injection of benzathine penicillin G.^24^ Timely treatment of syphilis in its early stages limits clinical progression and eliminates spread to partners through sexual contact. Early identification and treatment of syphilis are a priority goal of increased screening.

### Differences in Management and Outcomes

Once nearly eliminated, syphilis rates in the U.S. have steadily risen since the early 2000s and have recently reached historic heights. In countries that have implemented increased screening practices, the treatment and management of syphilis have improved. For example, an Australian study of a syphilis screening campaign for MSM launched in 2009 concluded that increased syphilis testing improved detection significantly for HIV-negative men (from 27% to 44%) and for HIV-positive men (from 23% to 45%).^25^ It also resulted in a relative reduction in progression to secondary syphilis (HIV-negative men’s rate decreased from 24% to 19%, and HIV-positive men’s rate decreased from 45% to 26%).^25^

How patients are screened also matters. The current national recommendations for syphilis screening use a risk-based approach whereby patients are screened only if they meet certain risk criteria. Identifying behaviors that may increase the risk of syphilis in clinical practice is a challenge that can lead to suboptimal screening. A national study in 2019 found that half of pregnant people with newly diagnosed syphilis reported no behavioral risks.^26^ In 2024, a study of young Black men who have sex with women in New Orleans, LA, found that only 19% of those meeting CDC risk-based screening criteria had ever been screened.^27^ Comparatively, population-wide, opt-out approaches make screening straightforward and streamlined for local providers to implement. It also removes the stigma associated with evaluating a patient for risks.^28^

Opt-out screening programs generate higher numbers of screened individuals and detection rates of active disease increase. This was demonstrated by the adoption of opt-out screening methods for HIV. A systematic review and meta-analysis of 150 studies published in 2022 determined that opt-out HIV screening methods resulted in the largest uptake of testing (64.3%) than opt-in (59.8%) and risk-based (54.5%) screening methods.^29^ In a 2024 NIH-funded study of opt-out syphilis screening in an urban emergency department (ED), an opt-out approach increased syphilis screening from 3.6% to 24.4% of all ED encounters.^30^ The diagnosis of presumed active syphilis infection (reactive treponemal antibody [IgG] and reactive rapid plasma reagin or *Treponema pallidum* particle agglutination without a documented history of past positive testing or treatment) increased by 288%, with the number needed to screen being approximately 75 individuals to detect 1 active infection.^30^

### Accuracy

The traditional syphilis screening algorithm involves an initial nontreponemal test (rapid plasma reagin or Venereal Disease Research Laboratory) followed by a confirmatory treponemal test (fluorescent treponemal antibody absorption or *Treponema pallidum* particle agglutination), although a reverse screening algorithm is acceptable, in which a treponemal test is performed initially. The reverse algorithm is recommended by MDH to detect early and late, untreated infections, but local factors should be considered when determining the clinical and laboratory approach to syphilis screening. Both approaches require a confirmatory test. The accuracy of different syphilis screening tests varies according to the stage of the disease.

According to review of the literature, nontreponemal tests range by 61%–78% for detecting primary and late latent syphilis and 85%–100% for detecting secondary and early latent syphilis.^31^ Sensitivity of treponemal tests range by 82%–100% across stages of the disease.^31^ Specificity of treponemal tests for confirming primary syphilis ranges by 94%–100%.^31^ There is limited evidence comparing the traditional and reverse sequence algorithms for syphilis screening. The reverse sequence algorithm can produce more discordant results that require additional testing than the traditional algorithm.^32^ It is also known that treponemal tests cannot differentiate between active and past-treated disease.^32^^,^^33^ Conversely, the traditional algorithm may have decreased sensitivity for detecting primary and latent syphilis.^32^^,^^33^ Therefore, both algorithms are acceptable and have their advantages and disadvantages.

### Other Considerations Derived from the GRADE Evidence-to-Decision Framework

There are no studies that directly compare the acceptability of risk-based, opt-in syphilis screening with that of population-wide, opt-out screening programs in the general population. There have been studies of high-risk populations that demonstrate acceptability. In Australia, a network of 46 sexual health clinics that primarily cared for a population of MSM demonstrated increased uptake of syphilis screening among HIV-negative MSM and HIV-positive MSM.^25^ In the previously cited study of opt-out syphilis testing in a large urban ED, uptake also increased with routine opt-out screening.^30^

Opt-out sexually transmitted infection screening programs have demonstrated feasibility in sexual health clinics, primary care, obstetrics clinics, and EDs.^29^^,^^30^^,^^34^ Two hospital EDs—one in Miami, Florida, and another in Cleveland, Ohio—demonstrated feasibility in implementing syphilis screening initiatives in the ED by bundling them with HIV/hepatitis C virus screening.^35^^,^^36^ Both programs favored the reverse algorithm because it is a quicker, more automated test, and both programs relied on collaboration with their local public health departments for linkage to follow-up care. An ED in Houston, TX successfully implemented an opt-out screening program for all pregnant individuals visiting the ED, which increased screening from 2% to 56.4%.^37^ With these updated recommendations, MDH encourages emergency medicine providers in the state to consider adopting screening initiatives in EDs and urgent care centers, which may serve as a primary access point to the health system for many patients.

The cost-effectiveness of syphilis screening in pregnant people has good economic evidence published in a systematic review last year.^38^ There have also been exploratory models to analyze the cost-effectiveness of syphilis screening in populations of high-risk MSM.^39^ However, no studies or models were identified that evaluate the cost-effectiveness of opt-out syphilis screening in nonpregnant adults. The Association of State and Territorial Health Officials published a report on policy considerations for reducing congenital syphilis in 2023, which included partnering with state Medicaid for support.^40^ Representatives from MDH met with Minnesota Medicaid and Minnesota healthcare partners to discuss implementation prior to releasing the updated recommendation. There was overall support from partners to address the rising syphilis rates in Minnesota. Future investigation into the cost-effectiveness of opt-out syphilis testing is needed.

Although rising syphilis rates have been recorded across all races, sexes, ethnicities, and sexual orientations, there has been a disproportionate impact among historically marginalized Black, Indigenous, and People of Color (BIPOC) communities and MSM. Thus, equity is a concern. BIPOC communities may be disproportionately impacted by syphilis owing to factors that influence the social determinants of health. Implementing population-wide, opt-out screening helps healthcare providers avoid promoting the stigma that may be associated with risk-based screening and associating high risk factors with BIPOC and MSM communities.^41^

Finally, screening for syphilis can be associated with plausible harms. There are false-positive results that lead to further testing, anxiety, and possible stigma. Biologic false-positive results may occur in up to 0.8% of the population when using the nontreponemal test.^33^ The reverse algorithm may result in more false positives when applied to low-prevalence populations as well as an increased number of false positives owing to prior syphilis infection in high-prevalence populations.^33^ Downstream harms can include those related to treatment, such as antibiotic resistance and antibiotic side effects, although greater harm may occur for those infected and left untreated.^42^ An additional consideration is the overutilization of benzathine penicillin G, which has greater implications during periods of shortages.

## FINAL RECOMMENDATION AND IMPLEMENTATION CONSIDERATIONS

With any screening test, there are clinical, ethical, and economic impacts to consider. Screening can result in false positives, which may lead to undue psychological distress.^43^ In a 2024 study, it was reported that women who had a false-positive screening mammogram that required repeat diagnostic testing were less likely to return to routine screening.^44^ An opt-out option should be offered and respected when it is elected by the patient.

An additional burden is placed on healthcare systems and providers to follow up on test results and link patients to care. Partner services through MDH offer help with partner notification and treatment. All confirmed cases of syphilis are reported electronically to MDH, and partners/contacts elicited during case interviews are notified of exposure and referred for medical evaluation. Untreated cases of all stages are linked to treatment or have treatment confirmed.^45^

**Figure 3:**
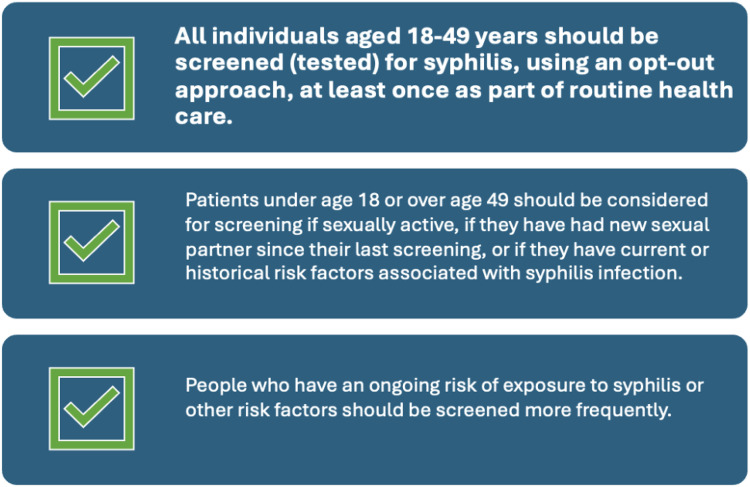
Final recommendation from MDH on syphilis screening. MDH, Minnesota Department of Health.

Minnesota healthcare providers should implement routine, opt-out screening for syphilis (Figure 3) in any healthcare setting, including primary care clinics, correctional facilities, and substance use treatment facilities. In addition, it should be considered in EDs or urgent care settings that treat high-risk populations or have high local rates of syphilis or for patients with limited healthcare access, especially pregnant patients. In these settings, it is important that there is a plan for follow-up and management of results. Although most private health insurance plans, plus Medicare and Medicaid, cover preventive services with a Grade A or B designation from the USPSTF, this is not necessarily the case for state recommendations. Healthcare providers should consider cost. The effectiveness of population-wide, opt-out testing for syphilis should be evaluated on an ongoing basis and refined on the basis of the uptake of screening, the local epidemiology of syphilis, and input from local healthcare providers.

## CONCLUSIONS

Increased screening is needed to combat syphilis, which is readily curable with antibiotics that reduce harmful sequelae, including the later stages of syphilis and congenital syphilis. In this paper, a modified decision-making framework is presented for addressing syphilis screening changes at the state public health level. The final, updated screening recommendation from MDH includes all sexually active people and populations. Other states are encouraged to evaluate the epidemiology of syphilis in their jurisdictions and consider reviewing and updating screening guidance on the basis of the proposed framework. Moving forward, universal, opt-out syphilis screening should be evaluated, including individual and community risk, benefit, and cost considerations.
